# Sacral incidence to pubis: a novel and alternative morphologic radiological parameter to pelvic incidence in assessing spinopelvic sagittal alignment

**DOI:** 10.1186/s12891-021-04093-z

**Published:** 2021-02-23

**Authors:** Yasuhito Takahashi, Kei Watanabe, Masashi Okamoto, Shun Hatsushikano, Kazuhiro Hasegawa, Naoto Endo

**Affiliations:** 1 Department of Orthopedic Surgery, Niigata Rosai Hospital, 1-7-12 Touncho, Joetsu City, 942-8502 Japan; 2 Department of Orthopedic Surgery, Niigata University Medical and Dental General Hospital, 1-757 Asahimachidori, Niigata City, 951-8510 Japan; 3 Niigata Spine Surgery Center, 2-5-22 Nishi-machi, Niigata City, 950-0165 Japan

**Keywords:** Pelvic incidence, Spinopelvic sagittal alignment, Hip dislocation, Femoral head deformities, Lumbar lordosis

## Abstract

**Background:**

Although pelvic incidence (PI) is a key morphologic parameter in assessing spinopelvic sagittal alignment, accurate measurements of PI become difficult in patients with severe hip dislocation or femoral head deformities. This study aimed to investigate the reliability of our novel morphologic parameters and the correlations with established sagittal spinopelvic parameters.

**Methods:**

One hundred healthy volunteers (25 male and 75 female), with an average age of 38.9 years, were analysed. Whole-body alignment in the standing position was measured using a slot-scanning X-ray imager. We measured the established spinopelvic sagittal parameters and a novel parameter: the sacral incidence to pubis (SIP). The correlation coefficient of each parameter, regression equation of PI using SIP, and regression equation of lumbar lordosis (LL) using PI or SIP were obtained. The intraclass correlation coefficient (ICC) was calculated as an evaluation of the measurement reliability.

**Results:**

Reliability analysis showed high intra- and inter-rater agreements in all the spinopelvic parameters, with ICCs > 0.9. The SIP and pelvic inclination angle (PIA) demonstrated strong correlation with PI (*R* = 0.96) and pelvic tilt (PT) (*R* = 0.92). PI could be predicted according to the regression equation: PI = − 9.92 + 0.905 * SIP (*R* = 0.9596, *p* < 0.0001). The ideal LL could be predicted using the following equation using PI and age: ideal LL = 32.33 + 0.623 * PI – 0.280 * age (*R* = 0.6033, *p* < 0.001) and using SIP and age: ideal LL = 24.29 + 0.609 * SIP – 0.309 * age (*R* = 0.6177, *p* < 0.001).

**Conclusions:**

Both SIP and PIA were reliable parameters for determining the morphology and orientation of the pelvis, respectively. Ideal LL was accurately predicted using the SIP with equal accuracy as the PI. Our findings will assist clinicians in the assessment of spinopelvic sagittal alignment.

**Trial registration:**

This study was retrospectively registered with the UMIN Clinical Trials Registry (UMIN000042979; January 13, 2021).

## Background

More than two decades ago, the concept of pelvic incidence (PI) was introduced, and it has become the significant unique parameter for assessment of standing spinal alignment [1]. For example, a large PI is associated with a great sacral slope (SS) and a pronounced lumbar lordosis (LL), and a low PI is associated with a smaller SS and a subtle LL, and these observations represent the basic concept of global sagittal alignment during standing [1–6]. Previous studies have reported that deterioration of sagittal alignment, especially loss of LL, decreases health-related quality of life (HRQOL) [7–11]. Schwab et al. reported that PI minus LL and pelvic tilt (PT) combined with the sagittal vertical axis (SVA) can predict disability, and they proposed threshold values of sagittal modifiers (PI-LL ≥ 10°, PT ≥ 20°, SVA ≥ 4 cm) for disability of HRQOL in the SRS-Schwab adult spinal deformity classification [12, 13]. Among them, PI minus LL mismatch has been used as the most essential therapeutic target, which can be directly controlled during corrective fusion surgeries.

Moreover, lumbar degenerative spondylolisthesis, a common degenerative disorder, which causes sagittal malalignment as well as disability of daily life, tends to exhibit a high PI [14, 15], and is associated with coexistence of osteoarthritis of hip [16, 17]. Recently, a consensus has emerged that restoration of sagittal spinopelvic alignment is the primary goal of surgical treatment for adult spinal deformity (ASD) [12, 18].

There is a reciprocal relationship between the lumbar spine and hip joint characterised by ‘hip-spine syndrome’, which was reported by Offierski and MacNab [19]. There are two types of pathologies in hip-spine syndrome. The first type includes primary hip lesions, such as hip dysplasia with compensatory pelvic anteversion causing spondylolisthesis or spinal canal stenosis developed by lumbar hyperlordotic alignment. Hip dislocation with pelvic obliquity can cause secondary lumbar scoliosis. The other type includes primary lumbar spinal lesions, such as spinal kyphosis with compensatory pelvic retroversion causing acute progressive hip arthritis. Therefore, it is essential that both hip and spine surgeons pay attention to whole body alignment, including the spine and lower extremities, and compensation mechanisms for deteriorating standing alignment with degenerative changes or aging [20]. In particular, PI is a key parameter in assessing sagittal morphology and pathology of the hip–spine, as well as guiding surgical decision-making [1, 18]. When measuring these parameters on a lateral view of the pelvis, it is critical to identify the hip axis (HA), which is represented by the line connecting the centres of the femoral heads [1]. However, in patients suffering from progressive hip arthritis with hip dislocation or femoral head deformity, accurate identification of the axis of the femoral heads is not possible.

Therefore, alternative morphological pelvic parameters need to be established. The upper edge of the pubic symphysis has also been proposed as a key anatomic landmark of the anterior pelvic plane and used in assessing pelvic orientation [21, 22]. We focused on establishing positional parameters of the pelvis using the upper edge of the pubic symphysis and designed our novel model of the morphologic parameters of the pelvis. We hypothesised that our new parameter could be used as an alternative pelvic morphologic parameter to PI. The purpose of this study was to investigate the reliability of these parameters and the correlations with established sagittal spinopelvic parameters in asymptomatic adults.

## Methods

This prospective observational study was approved by the Clinical Research Ethics Committee of the hospital. All Niigata Spine Surgery Center staff (physicians, nurses, technicians, pharmacists, medical clerks, etc.) were informed of the purpose of the study according to the Helsinki Declaration. After signing the informed consent form, anthropometric, clinical, and radiographic data were collected from those who agreed with the purpose of the study. One hundred and twenty-one volunteers with no history of treatment for spinal disease were enrolled. After whole spinal radiographic images were obtained, we excluded five cases with lumbarisation, three cases with sacralisation, one case with 11 thoracic vertebrae, two cases with scoliosis (Cobb angles > 20 degrees), three cases with lumbar spondylolisthesis with %slip of at least 5 %, three cases with axial pelvic rotation > 5 degrees, two cases with total hip arthroplasty or postoperation of the ilium, and two cases that moved during scanning, so that accurate radiographic measurements could be obtained. Exclusion of the transitional vertebrae is important because transitional vertebrae affect spinal and pelvic parameter measurements. Finally, 100 patients, including 25 males and 75 females, with an average age of 38.9 years (20–70 years), were included for radiographic analysis. The following epidemiologic and morphologic characteristics of patients were obtained: age, sex, body weight, height, and body mass index (BMI).

### Radiological measurements

Whole-body alignment in the standing position was measured using a slot-scanning X-ray imager (EOS Imaging, Paris, France). Patients were instructed to stand in the centre of the imager and look straight ahead at a horizontal gazing mirror, while placing their hands on their cheeks. The patients were scanned within 30 s from the skull to the feet at a speed of 7.6 cm/s [23]. The average dose of radiation was 23.3 mGy•cm^2^ including the front and side surfaces.

**Fig. 1:**
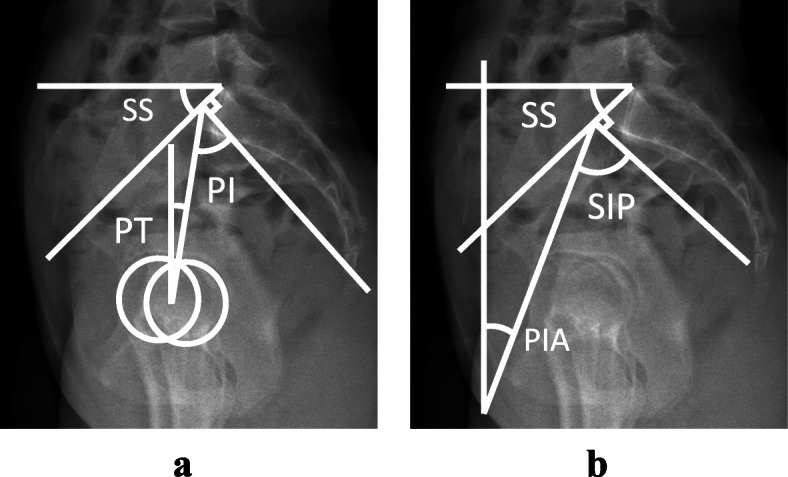
**a** Illustration of the conventional radiological parameters of the pelvis on the sagittal plane. The parameters illustrated include pelvic incidence (PI), pelvic tilt (PT) and sacral slope (SS). **b** Illustration of the novel radiological parameters of the pelvis on the sagittal plane. The parameters illustrated include sacral incidence to pubis (SIP), pelvic inclination angle (PIA) and sacral slope (SS)

We measured the PI, PT, SS, and LL (L1-S1) as routine spinopelvic sagittal parameters (Fig. 1a). Pelvic parameters have the following geometrical relationship: PI = PT + SS. In addition, we measured the following parameters: (1) the sacral incidence to pubis (SIP), which is the value of the angle between the line perpendicular to the superior plate of the first sacral vertebra (S1) at its anterior edge and the line connecting this point to the upper edge of the pubic symphysis and (2) the pelvic inclination angle (PIA) [22], which is the value of the angle between the vertical line and the line connecting the anterior edge of the sacral plate to the upper edge of the pubic symphysis (Fig. 1b). SIP is the novel parameter we defined, while PIA is a previously reported parameter [22]. These parameters have the following geometrical relationship: SIP = PIA + SS.

This time, we used the upper edge of the pubic symphysis as the centre of pelvic version instead of HA, and considered PIA as a positional parameter indicating pelvic version instead of PT. Therefore, our new parameter SIP is equal to the sum of SS and PIA, and there is a great similarity in the geometrical formula between PI and SIP.

Three authors independently and blindly measured 30 cases selected randomly from the 100 patients to compare inter-rater reliability. In addition, these measurements were repeated by the same three authors after more than 2 weeks to compare intra-rater reliability.

### Statistical analysis

In order to analyse the intra- and inter-rater reliabilities of the parameters, intraclass correlation (ICC) estimates and their 95 % confidence intervals were calculated based on a single-rating, absolute-agreement, 2-way mixed-effects model. The correlations between the investigated parameters were analysed by Pearson’s correlation coefficients. Simple linear regression analysis was performed to establish a formula to predict the value of PI using the newly-established anatomical parameter. In addition, we defined the LL of healthy volunteers with no history of treatment for spinal disorders who participated in this study as the ideal LL. Stepwise multiple regression analyses were performed to establish formulas to predict the ideal LL value using age, sex, height, weight and PI or SIP. As SS, PT and PIA are dependent factors affecting spinal deformity, we did not include them as independent variables. JMP (version 9; SAS Institute, Cary, NC) and SPSS (IBM SPSS Statistics for Windows, Version 24.0, IBM Corp., Armonk, NY) were used for the analyses, and a p value less than 0.05 was considered statistically significant.

## Results

Demographic and radiographic parameters are shown in Table 1. Distributions of all radiographic parameters, except for age and weight, were within the normal range.

**Table 1:** Demographic and radiographic parameters

	Mean	Range (min/max)	SD	SE	IQ 25 %/75 %
Age (years)	38.9	20/70	11.0	1.1	31.0/45.0
Weight (kg)	55.6	38/83	9.0	0.9	49.0/62.0
Height (cm)	161.8	146/180	7.8	0.8	156.0/167.8
BMI (kg/m^2^)	21.1	14.5/27.2	2.4	0.2	19.6/22.8
PI (°)	51.0	30.9/79.5	10.5	1.1	42.8/59.1
SIP (°)	67.3	47.0/102.2	11.2	1.1	59.0/74.5
SS (°)	40.4	15.3/61.5	8.6	0.9	35.7/46.8
PT (°)	10.7	–7.0/35.5	7.5	0.8	5.5/15.0
PIA (°)	26.9	10.7/52.0	7.8	0.8	21.4/32.2
LL (°)	53.3	24.8/77.7	11.1	1.1	47.1/60.3

*SD* standard deviation, *SE* standard error, *IQ* interquartile, *BMI* body mass index, *PI* pelvic incidence, *SIP* sacral incidence to pubis, *SS* sacral slope, *PT* pelvic tilt, *PIA* pelvic inclination angle, *LL* lumbar lordosis

For the radiographic measurements, the reliability analysis showed high intra- and inter-rater agreements for all the spinopelvic parameters, with ICCs > 0.9 (Table 2).

**Table 2:** Intra- and inter-rater reliability of the spinopelvic parameters

	Intra-rater reliability						Inter-rater reliability	
	Examiner A		Examiner B		Examiner C			
	ICC	95% CI	ICC	95% CI	ICC	95% CI	ICC	95% CI
PI	0.97	0.94–0.99	0.98	0.97–0.99	0.99	0.99–1.00	0.98	0.95–0.99
SIP	0.96	0.92–0.98	0.98	0.96–0.99	0.99	0.98–1.00	0.96	0.96–0.98
SS	0.95	0.89–0.97	0.98	0.95–0.99	0.99	0.98–1.00	0.95	0.91–0.98
PT	0.99	0.98–1.00	1.00	0.99–1.00	1.00	0.99–1.00	0.99	0.94–1.00
PIA	0.99	0.99–1.00	0.99	0.99–1.00	0.99	0.99–1.00	0.99	0.98–1.00
LL	0.96	0.92–0.98	0.98	0.96–0.99	0.99	0.99–1.00	0.96	0.92–0.98

*ICC* intraclass correlation, *CI* confidence interval, *PI* pelvic incidence, *SIP* sacral incidence to pubis, *SS* sacral slope, *PT* pelvic tilt, *PIA* pelvic inclination angle, *LL* lumbar lordosis

The correlations among radiographic parameters using a Pearson’s correlation analysis are shown in Table 3. High positive correlations [*correlation coefficient (R)* > 0.9] were found between PI and SIP, and PT and PIA. Moreover, the correlation coefficient between SIP and LL was similar to that between PI and LL. The same applied to the correlation coefficient between SS and PI, and that between SS and SIP (Table 3)

**Table 3:** Correlation coefficients among radiographic parameters

	PI	SIP	SS	PT	PIA	LL
PI	-					
SIP	0.96^*^	-				
SS	0.71^*^	0.72^*^	-			
PT	0.59^*^	0.53^*^	–0.15	-		
PIA	0.59^*^	0.64^*^	–0.07	0.92^*^	-	
LL	0.54^*^	0.54^*^	0.87^*^	–0.24^*^	–0.18	-

*PI* pelvic incidence, *SIP* sacral incidence to pubis, *SS* sacral slope, *PT* pelvic tilt, *PIA* pelvic inclination angle, *LL* lumbar lordosis

* *p* < 0.0001

### Prediction of PI

SIP was shown to be a predictor of PI according to the regression equation: PI = − 9.92 + 0.905 * SIP (*R* = 0.9596, *p* < 0.0001).

### Prediction of ideal LL

With a stepwise multiple regression analysis of an ideal LL, sex, height and weight were excluded, and age and PI or SIP were selected as contributing factors. Then we produced the following equation using PI and age:

1 $$ \mathrm{ideal}\ \mathrm{LL}=32.33+0.623\ast \mathrm{PI}-0.280\ast \mathrm{age}\ \left(R=0.6033,p<0.001\right). $$

We also produced another equation using SIP and age:

2 $$ \mathrm{ideal}\ \mathrm{LL}=24.29+0.609\ast \mathrm{SIP}-0.309\ast \mathrm{age}\ \left(R=0.6177,p<0.001\right). $$

## Discussion

In the present study, we investigated conventional and novel measurement patterns of spinopelvic sagittal alignment in healthy adult volunteers. The standing sagittal alignment was similar to that in previous studies. The correlations among the radiographic parameters of sagittal alignment were also comparable with those of previous reports [24, 25]. The correlation between SIP and SS, PIA, and LL is remarkably similar to the correlation between PI and SS, PT, and LL. Just as PI plays a key role in the regulation of positional pelvic and spinal parameters, SIP has the potential to be a key parameter in spinopelvic alignment [25].

As mentioned previously, the evaluation of spinal sagittal alignment, as well as pelvic version according to pelvic morphology, especially PI, are crucial in hip or spine surgeries. Although use of HA, which is the centre of pelvic version, is optimal to assess pelvic morphology, an accurate assessment of PI is impossible on lateral plane radiographs in cases with deformed or dislocated femoral heads due to severe osteoarthritis or necrosis. Recently, alternative morphologic parameters have been proposed, without use of HA. Imai et al. reported a strong correlation between PI and anatomical-SS as a morphological parameter, which is the value of the SS measured from the pelvis adjusted in the anterior pelvic plane [26]. Wang et al. suggested sacrum pubic incidence as another alternative parameters with strong correlation with PI for determining the morphology of the pelvis [24]. Instead of the HA, the upper edge of the pubic symphysis has been proposed as a key anatomic landmark of the anterior pelvic plane for assessing pelvic orientation, and we have followed a similar concept with our novel parameter. Although the superior plate of the S1 at its midpoint is identified as a bending point in the traditional parameters, identification of the posterior edge of S1, which is needed to find the midpoint, is difficult to identify in some cases with strong L5/S1 degeneration. Therefore, we used the superior plate of the S1 at its anterior edge as a bending point for our novel parameter, SIP, which does not require the posterior edge of S1. Furthermore, the PIA can be used to evaluate pelvic orientation instead of the PT [22]. In the present study, both SIP and PIA showed very high intra-observer and inter-observer reliability (all ICCs > 0.95), and strong correlation with PI and PT, respectively (both *R* > 0.9). Therefore, these two parameters could be alternative morphologic or positional parameters of the pelvis to assess whole body sagittal alignment.

Previous studies have reported that deterioration of sagittal alignment, especially loss of LL due to common spinal disorders, decreases HRQOL [7–11]. Therefore, the purpose of reconstructive surgery is to restore physiological LL and PT, which is equivalent to approximating LL to PI [12, 18]. Although PI is a crucial target during reconstructive surgery, accurate identification of PI is not possible in patients suffering from hip-spine syndrome due to deformed or dislocated femoral heads. Therefore, the alternative morphological pelvic parameter that we propose and our novel concepts for prediction of ideal LL are useful for evaluation of lumbar degeneration as well as alignment correction planning in such complex cases during reconstructive surgery. There have been several concepts related to the prediction of the ideal LL, and the lordosis predictive equation can be based for not only age and pelvic morphologic parameters, but also for thoracic kyphosis and L1 or T9 tilt because of the reciprocal chain of correlations between the positional pelvic and spinal parameters and the morphological PI [25, 27]. However, since positional parameters, especially spinal parameters, can be frequently affected by common aging conditions, such as spinal fractures and degenerative disc diseases, our simple equation using a definitive morphologic parameter to predict ideal LL would be practical in the clinical setting.

Two limitations of our study should be acknowledged. Firstly, the proportion of female volunteers we recruited in this study was as high as 75 %. However, a previous report showed that sex has no effect on spinopelvic parameters [25]. Secondly, the study was conducted on individuals with a wide age range, in which age-related degeneration may have affected spinopelvic alignment in some cases. Although our previous study with 136 healthy patients showed that there was no statistically significant correlation between age and LL [20], LL decreases slightly with age [28]. Therefore, age was selected as a contributing factor for ideal LL in our study. Even if participants with a wide age range are analysed, the strong correlation between SIP and PI can be said to be applicable to cases of any age.

On the other hand, the present study has some strong points compared to the previous studies. Firstly, we investigated sagittal whole-body skeletal alignment and used a scanning X-ray imager with a biplanar upright scanning imaging modality to achieve reduced X-ray particle scatter, improved image quality and significantly reduced radiation to the patient [23]. Secondly, we strictly excluded all patients with anomalous vertebrae, such as transitional vertebrae and scoliosis with a Cobb angle > 20°, which can affect measurement precision.

From the perspective of future possibilities, we need to investigate the accuracy or reliability of SIP and PIA measurements on conventional X-ray images compared to PI and PT. Moreover, further studies are needed on whether our novel concepts can predict the HRQOL status in patients with progressive hip arthritis with hip dislocation or femoral head deformity. We believe that use of our novel concepts can support future research in the elucidation of the various pathologic conditions on hip-spine syndrome.

## Conclusions

Since the surgical goal is to achieve optimal global sagittal alignment by restoring an optimal LL for spinal deformity patients to enhance quality of life status, our novel pelvic parameters accurately assessed spinopelvic sagittal alignment, and allowed us to create formulae for predicting PI and an ideal LL. Our new measurement parameters and formulae may assist clinicians in treatment decision-making, and lead to better quality of life for patients with ASD and those with progressive disorders of the hip joint.

## Data Availability

The data analysed during the current study are available from the corresponding author on reasonable request.

## Abbreviations

PI: Pelvic incidence
SS: Sacral slope
LL: Lumber lordosis
HRQOL: Health-related quality of life
PT: Pelvic tilt
SVA: Sagittal vertical axis
ASD: Adult spinal deformity
HA: Hip axis
BMI: Body mass index
SIP: Sacral incidence to pubis
S1: The first sacral vertebra
PIA: Pelvic inclination angle
ICC: Intraclass correlation
R: Correlation coefficient
